# Ubiquitinations in the Notch Signaling Pathway

**DOI:** 10.3390/ijms14036359

**Published:** 2013-03-19

**Authors:** Julien Moretti, Christel Brou

**Affiliations:** 1 Immunology Institute, Department of Medicine, Mount Sinai School of Medicine, 1425 Madison Avenue, New York, NY 10029, USA; E-Mail: julien.moretti@mssm.edu; 2 Institut Pasteur and CNRS URA 2582, Signalisation Moléculaire et Activation Cellulaire, 25 rue du Docteur Roux, 75724 Paris cedex 15, France

**Keywords:** Notch, ubiquitination, deubiquitinating enzyme, signal transduction, trafficking

## Abstract

The very conserved Notch pathway is used iteratively during development and adulthood to regulate cell fates. Notch activation relies on interactions between neighboring cells, through the binding of Notch receptors to their ligands, both transmembrane molecules. This inter-cellular contact initiates a cascade of events eventually transforming the cell surface receptor into a nuclear factor acting on the transcription of specific target genes. This review highlights how the various processes undergone by Notch receptors and ligands that regulate the pathway are linked to ubiquitination events.

## 1. Introduction

Development and maintenance of organs and tissues requires constant interaction and communication between cells. One of the major signaling pathways involved in cell-cell communication is the Notch pathway. This pathway is highly conserved in animals as distantly related as *C. elegans*, *Drosophila*, and vertebrates, with regard to its roles as well as to its molecular mechanisms and factors involved. As a matter of fact the Notch gene has been first identified in *Drosophila*[1] and T. Morgan had already described the outcome of Notch haploinsufficiency, the presence of notches at the edge of fly wings. Notch signaling pathway is still intensively studied in these organisms with the benefit of powerful genetic tools.

Notch activation is initiated when a Notch receptor-expressing cell directly contacts a neighboring ligand-expressing cell. This way of communication between adjacent cells leads to many downstream responses, including cell-fate specification, progenitor cell maintenance, boundary formation, cell proliferation and apoptosis. This pathway is used at many steps during development and adulthood, and the precise outcome of the Notch signal is highly sensitive to the cellular context. Haploinsufficiency or gain-of-function of *Notch*, or *Notch*-related genes, are responsible for various human pathologies: developmental diseases, including aortic valve diseases, Alagille syndrome, and familial forms of cardiomyopathy, or late-onset syndromes, like CADASIL (Cerebral Autosomal Dominant Artheriopathy with Subcortical Infarcts and Leukoencephalopathy [2]), a number of solid cancers (skin, intestine, head and neck squamous cell carcinoma, [3,4]) or leukemia (more than 50% of T-cell acute lymphoblastic leukemia T-ALL, 15% of chronic lymphocytic leukemia [5], myeloid leukaemia [6]. Depending on the tissue, *Notch* appears to behave as an oncogene or a tumor suppressor.

Notch signal transduction is unconventional first because it happens upon cell-cell contact, second because the membrane receptor itself is transformed into a transcription factor. In addition, the Notch pathway is unusual in that both ligands and receptors are affected by post-translational events that regulate their quantity, quality or even the activation process itself. These post-translational regulations include proteolysis (furin-processing of the receptor in the TGN, or successive cleavages by ADAM and gamma-secretase of the Notch receptor upon activation), unusual glycosylations of the receptor during its maturation [7], trafficking and finally modifications by ubiquitin attachment.

In this review we will describe the various steps where ubiquitination events affecting both the Notch receptors and their ligands are involved, and try to demonstrate how important these steps are.

## 2. Main Features of Notch Signal Transduction

**Figure 1:**
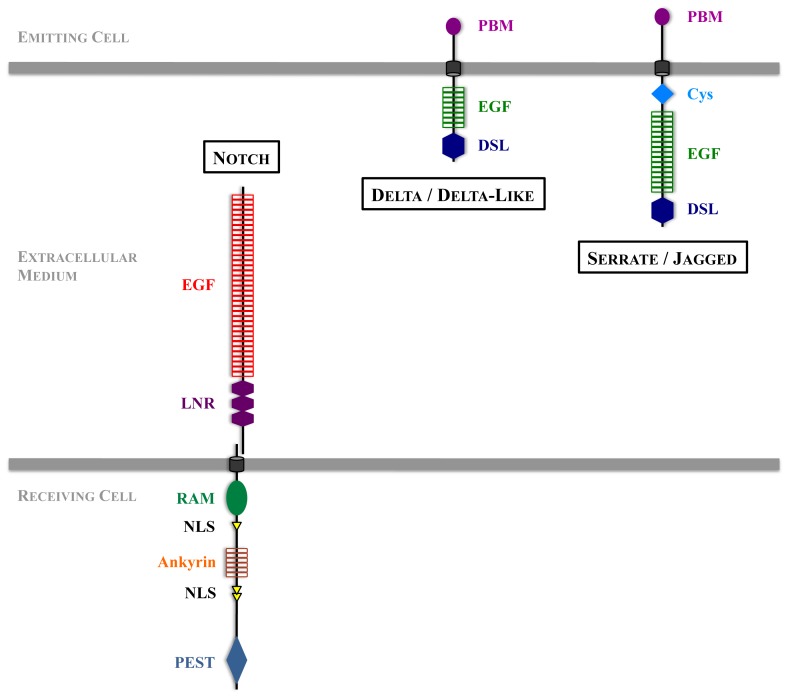
Structure of Notch receptor and DSL ligands. EGF: Epidermal Growth Factor-like repeats; LNR: Lin-12 Notch Region; RAM: RBPJ-Associated Molecule; PEST: Proline (P), Glutamic Acid (E), Serine (S) et Threonine (T); PBM: PDZ Binding Motif; DSL: Delta, Serrate, Lag-2; Cys: Cysteine.

Notch receptors are type I transmembrane proteins (see Figure 1) synthesized as proforms which are glycosylated, then cleaved few amino acids *N*-terminal to the transmembrane domain in the Trans-Golgi Network by a furin convertase, yielding an *N*-terminal extracellular fragment and a *C*-terminal transmembrane fragment. These two fragments associate as heterodimers and are eventually expressed at the cellular surface [8,9]. There are four Notch isoforms in mammals (for only one *Drosophila* Notch, Table 1), which all exhibit the same overall structure (Figure 1): EGF-like repeats and LNR domain (for Lin12-Notch-repeats) in the extracellular domain, and in the intracellular domain, several ankyrin-like repeats, a PEST domain (rich in proline, glutamic acid, serine and threonine residues), NLSs (nuclear localization signals), and a CSL-binding domain called RAM (CSL is for CBF1, Suppressor of Hairless, LAG-1, CSL is also termed RBPJκ, and RAM is for RBPJ/CSL association module). Notch ligands are also type I transmembrane proteins (Figure 1), containing extracellular EGF-like repeats, a DSL (for Delta-Serrate-Lag-2) motif accounting for Notch interaction, and short and quite divergent intracellular domains.

In mammals there are five canonical Notch ligands: Delta-like (Dll) 1, 3, 4 and Jagged 1 and 2, respectively homolog to their *Drosophila* counterparts Delta and Serrate (See Table 1 and Figure 1). In addition, noncanonical ligands have been described, either secreted or membrane-tethered proteins [10]. The way the activation proceeds does not seem to vary from one receptor or ligand to another. It is the combination of receptor/ligand as well as the cellular context that direct a specific response, and a better knowledge of how these variations are sensed by the cell will be necessary to understand the variety of pathological effects observed in case of overactivation or inhibition of the pathway. Whereas Notch pattern of expression is extremely wide (when taking into account the four genes in mammals, however each paralog has a specific pattern), the ligands may exhibit less redundancy in the area of expression and associated functions. For example, Dll4 and Jagged1 in mammals are prominent for angiogenesis and vasculature formation [11,12], Jagged1 for hepatic and biliary cells development [12], Dll1 and Jagged2 for somite and skeletal development [13,14], while Dll4 and Jagged2 have also functions in the thymus [14–16]. In addition Dll3 is proposed to be unable to activate Notch, but rather to alter Notch signaling levels that were induced by other DSL ligands, in particular during somitogenesis [17].

**Figure 2:**
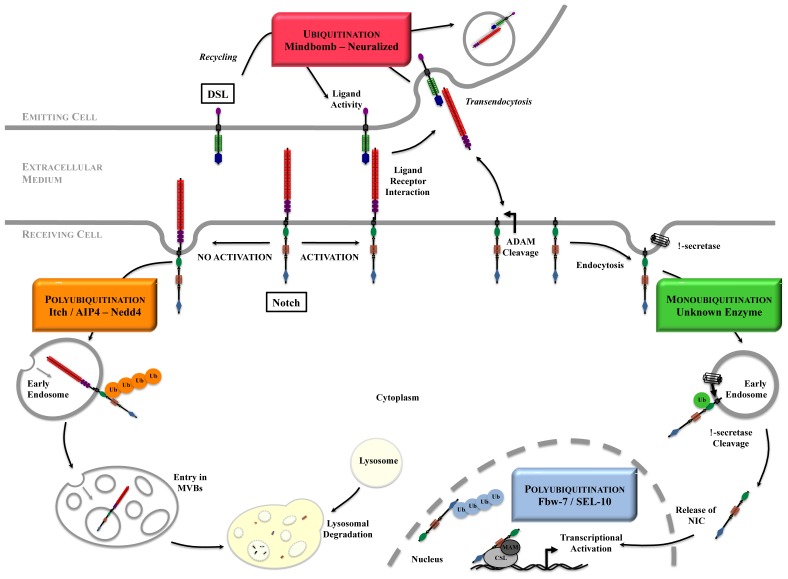
An overview of Notch signaling. The steps requiring ubiquitination are highlighted. In the absence of activation, Notch is internalized and degraded in the lysosomes. DSL ligands activity depends on trafficking and ubiquitination. Upon ligand binding, Notch extracellular domain is transendocytosed into the ligand-expressing cell, and the remaining receptor undergoes proteolytic cleavages as well as ubiquitination and deubiquitination to become a transcription factor.

Canonical Notch activation (that we restrict our review to) relies on two consecutive cleavages of the receptor initiated after ligand binding. Constitutive enzymes perform these maturations, however their accurate substrates are provided only upon activation. The first enzyme is an ADAM protease (A disintegrin and metalloproteinase, [18]): ADAM10 most of the time [19,20], ADAM17 in some contexts [21,22]. Notch receptor becomes an ADAM substrate when it binds to its ligand, probably because endocytosis of Notch extracellular domain (remember that Notch is an heterodimer) together with its ligand in the ligand-expressing cell separates Notch heterodimer and unmasks the ADAM cleavage site. After ADAM processing, Notch remains a transmembrane molecule, but amputated of most of its extracellular part, and becomes a gamma-secretase substrate. As it is the case for its other substrates (APP, E- and N-cadherins, ErbB-4, CD44 [23]), gamma-secretase acts inside Notch transmembrane domain and liberates the intracellular part of Notch from the membrane. The resulting product (Notch intracellular domain, hereafter named NIC) translocates to the nucleus, where it converts the DNA-binding protein CSL (also called RBPJ) from a transcriptional repressor into an activator. A ternary complex composed of CSL, NIC and one member of the Mastermind-like (MAM) family of coactivators is formed, and serves as a platform for recruitment of further coactivators, leading to transcriptional activation of Notch target genes (see Figure 2) [8].

The most classical target genes of Notch belong to the *HES* (hairy enhancer of split) and *Hrt* (Hes-related) families and encode for bHLH (basic helix-loop-helix) motif-containing transcription factors. These regulators generally repress transcription of genes involved in differentiation processes. As Notch signaling is associated with different outputs, depending upon the tissue, context-specific target genes may also be activated, in addition to the common targets like *HES* genes. For example, Notch activation may promote proliferation and maintenance of progenitor populations (e.g., myogenesis [24,25]), whereas in other tissues it allows cell type differentiation (e.g., T-cell formation [26] or neural fate [27]). Various genes including *c-myc*, *cyclinD*, *CDK5*, *EPHB2* or *EGFR*, or even Notch signaling regulators (*Deltex1*, *Notch3*) are described as Notch target genes in some context [28].

In contrast to other signaling pathways, where a cascade of second messengers is at stake, the activated Notch receptor is itself transformed into a transcriptional activator, NIC. Each Notch-responsive promoter may differ in the threshold of NIC required to activate it (depending on the target sequences, on their number and localization in the promoter); as a consequence, affecting NIC production and quantity directly affects Notch-dependent response. It has been shown in various systems that indeed perturbations of the efficiency of the overall process of signal transduction are at the origin of diseases [29]. Therefore, regulating this pathway means controlling spatiotemporal production and maintenance of active receptors and ligands at the cell surface, efficiency of signal transduction and stability of NIC. These key steps all involve ubiquitination events, that can thus influence the dosage-dependence and tissue context- or cell type-dependence of Notch signaling. Dysregulations of these events might be involved in various pathological processes in which the Notch signaling is disrupted.

## 3. Ubiquitination, Deubiquitination

**Figure 3:**
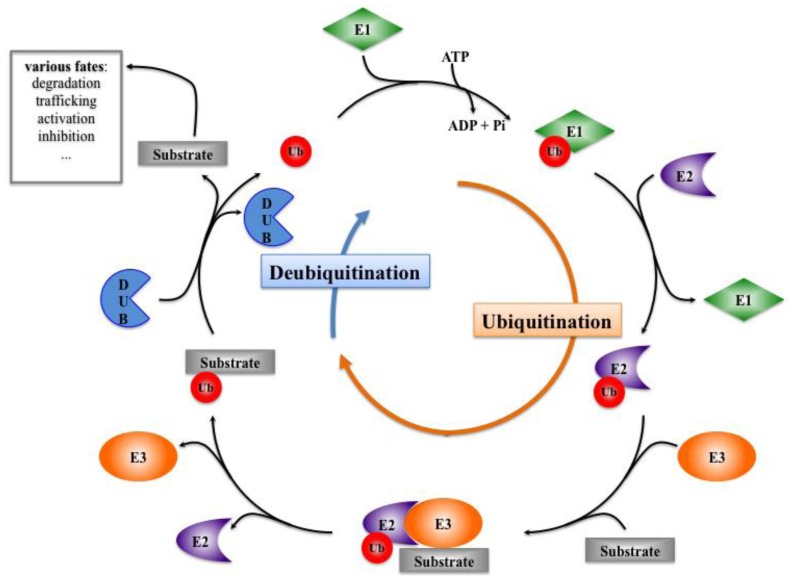
Ubiquitination and Deubiquitination processes. These processes allow ubiquitin to be recycled, whereas the substrates undergo various fates. Ub: Ubiquitin; E1: Ubiquitin Activating Enzyme; E2: Ubiquitin Conjugating Enzyme; E3: Ubiquitin Ligase; DUB: Deubiquitinating Enzyme.

Ubiquitin is a polypeptide of 76 amino acids, encoded by several genes in the form of a linear polyubiquitin precursor, which is processed by deubiquitinating activities into free ubiquitin molecules. Ubiquitin can be conjugated to a substrate by the formation of an isopeptide linkage between its last glycine residue and an internal lysine of the substrate (although alternative target amino acids have been described [30]). After conjugation of this first ubiquitin to the substrate, polyubiquitin chains can be elongated through internal lysine residues of ubiquitin molecule. Depending on which ubiquitin is used (lysine 6, 11, 27, 29, 33, 48, 63 of ubiquitin), the formed chains adopt various topological conformations and constitute specific signals that target the substrates to their downstream fate. The most popular chains are linked through Lysine 48 of ubiquitin, and target the substrates to proteasomal degradation [31]. As an additional layer of complexity, a given molecule can be modified by a single ubiquitin on a single lysine (monoubiquitination), by single ubiquitins on various lysines scattered over the substrate (multiubiquitination) or by ubiquitin chains on one or several lysines (polyubiquitination). These various modifications affecting a given substrate constitute signals that lead the molecules to a large panel of fates, including trafficking, activation, inhibition, or lysosomal degradation (see Figure 3).

Ubiquitin conjugation (or ubiquitination) depends on an enzymatic cascade involving E1 Ubiquitin-activating enzymes, E2 Ubiquitin-conjugating enzymes and E3 ubiquitin ligases (Figure 3). The E1 carries out the ATP-dependent activation of the *C*-terminus of ubiquitin and forms a covalent intermediate where the terminal glycine of ubiquitin is linked to the thiol group of a cysteine residue in the E1 active site. Ubiquitin is then transferred to the active cysteine residue of the E2, and eventually the E3 catalyzes the transfer of ubiquitin to a lysine residue in the protein substrate.

E3 ubiquitin ligases are the only enzymes in the ubiquitination process to be specific for the substrate, and they sometimes require cofactors to be recruited to their target. E3s belong to two large families, the HECT (Homologous to E6AP carboxyl terminus) and the RING (Really Interesting New Gene) that differ in the catalytic site and activity: whereas RING E3 ubiquitin ligases allow the transfer of ubiquitin from the E2-conjugating to the substrate, HECT enzymes conjugate the ubiquitin to their own active-site cysteine before transferring it to the substrate [32].

Much like what happens for phosphorylation, ubiquitination is counteracted by deubiquitination, which corresponds to the removal of the ubiquitin chain by a specific enzyme, called deubiquitinating enzyme or DUB. This deubiquitination step can either positively or negatively regulate the process initiated by ubiquitination. Therefore, for one ubiquitination event, two DUBs can be involved, one impairing ubiquitination and pulling back the process, the other allowing the ubiquitin signal to be transduced to the appropriate machinery.

DUBs belong to two main classes of proteases, Cysteine proteases and metalloproteases, and are divided into five subfamilies, based on their sequence similarities and likely mechanisms of action: UBPs (Ubiquitin-specific proteases), UCHs (Ubiquitin-carboxy-terminal hydrolases), OTUs (ovarian tumor), and Ataxin/Josephins harbor cysteine and Histidine-containing boxes in their catalytic domain, whereas JAMM/MPN+ proteases are Zinc-dependent metalloproteases [33,34].

There are less than 100 DUBs in the mammalian genome, 45 in the *Drosophila* genome [35]. In consideration of the large number of substrates (and even of the E3 ubiquitin ligases, almost 1000 in the human genome), it is obvious that each DUB probably recognizes several substrates, consisting in ubiquitinated molecules, or association of ubiquitinated substrates/type of ubiquitin chains/cofactors/E3 ubiquitin ligases. Because of this flexibility, genetic approaches at the level of organism are not favored to assign specific roles to DUBs in signaling pathways, although some clues have been recently obtained [36]. However despite this apparent lack of specificity, they are potential therapeutic targets with recent promising examples [36–39].

## 4. How Ubiquitinations Regulate Notch Pathway

### 4.1. NIC Stability

**Figure 4:**
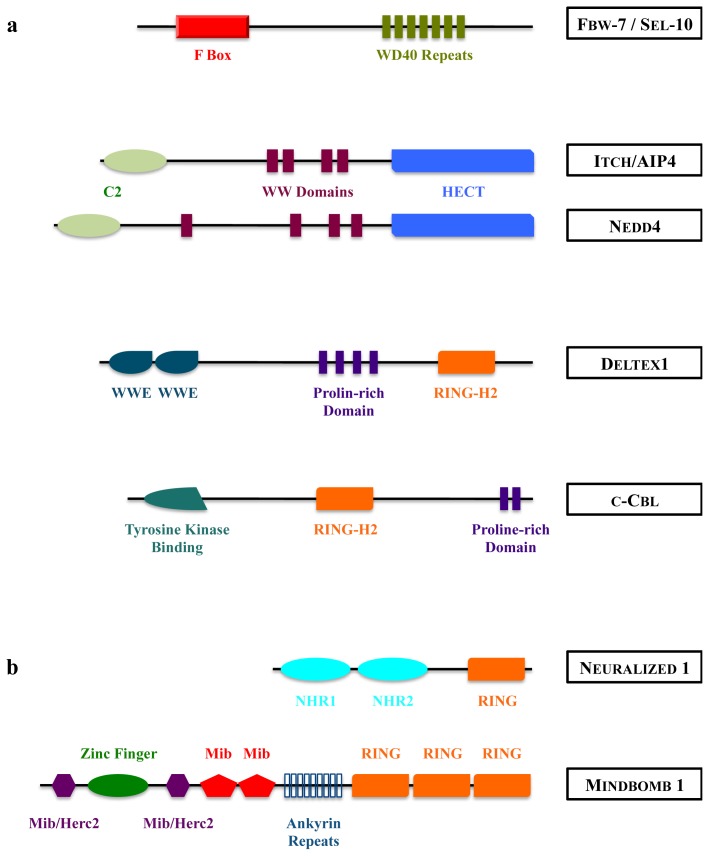
Structure of mammalian E3 ubiquitin ligases acting on Notch receptor (**a**) or DSL ligands (**b**). HECT: Homologous to E6-AP Carboxy Terminus; RING: Really Interesting New Gene; NHR: Neuralized Homology Repeat; Mib: Mindbomb; Herc2: HECT domain and RCC1 domain protein 2.

The first ubiquitination step identified in Notch signaling concerns the regulation of NIC stability. Notch intracellular domains exhibit canonical PEST domains (rich in proline, aspartic acid, serine and threonine residues), which are associated with short lifetime molecules. It has been demonstrated that NIC is indeed rapidly polyubiquitinated and degraded through the proteasomal pathway, and the E3 ubiquitin ligase accounting for these modifications is Fbw-7 (F-box and WD repeat domain-containing 7, see Figure 4 and Table 1) in mammals [40–42], its ortholog SEL-10 in *Caenorhabditis elegans*[43] and Archipelago in fly [44]. This enzyme belongs to the family of SCF (Skip1, Cul1, F-box) E3 ligases. In the SCF complex, Fbw-7 interacts on one side with the ubiquitination machinery via its F-box domain and on the other side provides substrate specificity by recognizing target proteins via its WD40 repeats-domain [45,46]. In addition to NIC, Fbw-7 is involved in the degradation of several oncoproteins, including c-myc, cyclin-E, c-Jun and NIC. It recognizes the substrates that harbor a high-affinity binding site called a Cdc4 phosphodegron (CPD), where two amino acids (threonine and serine) are phosphorylated [47]. In the case of NIC, Sel-10 recruitment to Notch was shown to require CDK8-dependent Notch phosphorylation [48].

This regulation of NIC by ubiquitination and proteasomal degradation is absolutely critical for an appropriate Notch signaling, since maintaining Notch signaling over a long period leads to severe pathologies: this is what happens in some T-ALL leukemia associated either with deletions in the *Notch1* gene, leading to Notch molecules deleted of their PEST domain, or with mutations in the *Fbw-7* gene, encoding an inactive or absent enzyme [47]. Besides, mutations of the gene encoding Fbw-7 are associated with many different cancers in human patients as well as in mouse models, implicating it as an important tumor suppressor [49].

Recently, Notch nuclear interactome was established using tandem affinity chromatography followed by mass spectrometry, in a human T-ALL cell line, where the endogenous constitutively activated Notch was inhibited by treating the cells with a gamma-secretase inhibitor and replaced by a tagged NIC. Among the interacting partners of NIC, several E3 ubiquitin ligases, including Fbw-7, and a limited number of DUBs, namely USP7, 11 and 15 were identified [50]. One can thus speculate that a lot remains to be understood in NIC regulation, linked to its stability, transport or associated factors.

### 4.2. Notch at the Cell Surface

It has been shown long time ago, in mammals as well as in *Drosophila* and *C. elegans*, that endocytic events affecting the receptor and the ligands are necessary in order for the pathway to be functional [51,52]. With regard to the Notch receptor, such events can regulate the quantity and quality of the Notch receptor at the surface of the cell but also the Notch activation process (Figure 2). Notch has a limited half-life at the cell surface and is constantly internalized, then recycled [53] or degraded through a lysosomal pathway, even in the absence of ligand [54–57]. This mechanism is a way of maintaining a functional receptor, and of eventually regulating Notch signal strength by acting on the receptor level at the cell surface.

Upon ligand activation, Notch receptor is also internalized and traffics into the endocytic pathway until being cleaved by gamma-secretase activity. Nevertheless the absolute requirement of these steps for Notch activation is still controversial, and some authors have proposed that gamma-secretase can process Notch at the cell surface ([58] and see below). Nevertheless most of the time, should it bind to its ligand or not, Notch initiates a journey through its internalization in early endosomal vesicles, which fuse then with early endosomes (EE). At this step, a cargo is normally either recycled back to the membrane through recycling endosomes, or remains in the maturing endosomes (ME). These ME are transformed into Multivesicular bodies (MVBs) through pinching off their limiting membrane as intraluminal vesicles (ILVs). Depending on whether the cargo is incorporated into these ILVs or stays at the limiting membrane of MVB, its fate will be different: degradation after fusion of the MVBs with the lysosomes in the first case, recycling in the second case. The formation of the ILVs is a central event that ensures a separation of the intracellular domain of the receptor from the cytosol, and thus potentially cancellation of the signal. The formation of the MVBs is controlled by the ESCRTs (Endosomal sorting complexes required for transport), which are macromolecular protein complexes acting sequentially and eventually allowing formation of the ILVs [59]. Cargo ubiquitination has been shown to be required for effective recruitment of the ESCRT machinery onto endosomal membrane and for the subsequent formation of ILVs [60]. ESCRT-0 initiates the pathway by engaging the ubiquitinated cargo. ESCRT-I and ESCRT-II complexes bind the cargo and each other to create an ESCRT-cargo-enriched zone, then ESCRT-II nucleates ESCRT-III assembly, which recruits the deubiquitination machinery and packages the cargo into the maturing vesicle, which is finally sorted upon vesicle budding [59]. Inactivating ESCRTs I, II or III [61–64], or other cofactors (for instance *lgd*[65]) in *Drosophila* leads to phenotypes resembling Notch activation, concomitantly to an accumulation of Notch molecules into enlarged endosomes. Indeed, defective ESCRT pathway maintains ubiquitinated Notch receptor at the limiting membrane of the MVBs (which contains gamma-secretase) and leads to ectopic and ligand-independent activation of Notch, therefore illustrating the crucial aspect of ILVs formation. In contrast, inactivating ESCRT-0 (*Hrs*) or early endocytosis modulators (*rab5*, *dynamin*, *syntaxin 1* for example) inactivates Notch signaling, in accordance with these steps being common for both Notch activation and degradation [62,63]. Peculiarly the ligand-independent activation of Notch when its degradation is impaired has never been observed in mammals, in contrast to the *Drosophila* system, maybe because alternative pathways are more efficiently used in mammals. During maturation, the lumen of the endocytic vesicles acidifies through the activity of vacuolar ATPase (vATPase), which has been shown to be necessary for ligand-dependent activation of Notch signaling in *Drosophila* and mammals [66]. This requirement was puzzling with regards to the documented late role of vATPase in the degradative pathway, however recent evidence suggests that vATPses are required for clathrin-mediated endocytosis by stimulating cholesterol recycling from endosomes back to the plasma membrane and therefore reducing the energy needed to constrict the neck of clathrin coated pits [67]. Together, these genetic data strongly support a crucial role for endocytosis of cell surface Notch as regulating the activation process as well as the half-life of the receptor.

Is Notch trafficking linked to its ubiquitination state, and does ubiquitination participate in discriminating activated from non-activated Notch?

Ubiquitination and trafficking are generally intimately linked processes, although the first event undergone by Notch is still unclear. Notch has no obvious internalization motif in its primary sequence able to direct its internalization. Whether ubiquitination at the cell surface, or perhaps other event, is involved in these first steps remains to be discovered. However, the later events are much better understood.

As often with this pathway, the start point of the discoveries was the identification by genetic means in *Drosophila* of several E3 ubiquitin ligases modulating Notch signaling activity, and the function of which begins to be understood.

Among them, Su(dx) (Suppressor of Deltex, Itch/AIP4 in mammals), Nedd4 (Neural precursor cell expressed developmentally down-regulated protein 4) belong to the HECT family, whereas Deltex (dx) is a RING-type E3 ubiquitin ligase (Figure 4a).

*Suppressor of deltex* (*Su*(*dx*)) was first described in *Drosophila* as acting in an antagonist manner to *Deltex* (*dx*), itself described by Morgan as a positive regulator of Notch signaling [68]. The phenotypes resulting from the overexpression of the Su(dx) protein in the developing wing are those expected from a downregulation of the Notch pathway. Mammalian orthologs of *Su(dx)* are called *Itch* in the mouse and *AIP4* (Atrophin 1-interacting protein 4) in humans. Mammalian *Itch* was identified in natural mutant mice (itchy mice, [69]) that develop a progressive autoimmune-like disease partly because Itch targets such as junB are relevant to autoimmunity, partly because of increased Notch signaling in the absence of *Itch*[70]. In mammals the non-activated Notch receptor is constitutively internalized, then ubiquitinated by Itch/AIP4 E3 ubiquitin ligase in order to be addressed to lysosomal degradation [54]. Interestingly the type of ubiquitin chain formed on Notch is proposed to be through Lysine 29 of ubiquitin, which is an unusual type of linkage [54]. In *Drosophila*, Su(dx) or/and Nedd4 fulfill the same function, regulating the postendocytic sorting of Notch en route to the late endosome by promoting its ubiquitination [55,57]. Whereas in mammals, Notch receptors are unable to directly bind HECT E3 ubiquitin ligases, *Drosophila* Notch exhibits in its intracellular domain a canonical PPXY motif accounting for such a direct interaction [54,55]. It is therefore possible that additional factors are necessary for the degradation complex formation and stability. The adaptor protein Numb could fulfill part of this function, since it cooperates with Itch to promote late sorting events leading to Notch 1 or 2, but not Notch 3 ubiquitination and degradation [53,71,72]. In *Drosophila*, the E3 ubiquitin ligase Deltex, together with Krz (the unique homolog of β-arrestins) and shrub (homolog to CHMP4, one of the ESCRT-III proteins) have been proposed to regulate the ubiquitination status of Notch via the endosomal/lysosomal pathway [73,74]. It remains to be demonstrated how these various factors interact with each other, if alternative ways exist for Notch to be degraded and how conserved are these mechanisms.

Recently, our work led to the identification of the deubiquitinating complex USP12/UAF1, which is recruited by Itch to non-activated Notch and allows Notch deubiquitination, probably before its entry into the intraluminal vesicles of the MVBs [75]. USP12 is therefore necessary for the accomplishment of Notch degradation. Probably by hijacking Notch degradative trafficking towards the recycling pathway, USP12 invalidation results in an increased quantity of receptor at the cell surface and consequently to a higher Notch response. Notably, a loss-of-function of *USP12* homolog in *Drosophila* leads to a phenotype reminiscent of a gain-of-function of Notch, strongly suggesting that not only the E3 ubiquitin ligases and the ubiquitination events, but also the DUBs, are conserved along evolution.

A puzzling E3 ubiquitin ligase involved in Notch signaling is Deltex. Genetic analysis in *Drosophila* supports the idea that Deltex (the product of the *dx* gene) is a positive regulator of Notch signaling [76]. *Drosophila dx* gene is required for the presence of N in endocytic vesicles [77,78], and for the full activity of Notch in a subset of developmental contexts. However in mammals, Deltex has been shown to act either positively or negatively, depending on the cellular context [79–81]. The presence of several genes and isoforms (4 members of the family have been identified in mammals [80]) may explain why gene deletion analyses were not conclusive. Deltex proteins contain *N*-terminal WWE domains accounting for direct interaction with Notch [82,83], a proline-rich domain, and a *C*-terminal RING domain, accounting for E3 ubiquitin ligase activity of Deltex isoforms [84,85]. On a biochemical level, Deltex has been shown in different systems to interact with factors promoting Notch degradation (shrub and arrestin-like complexes in *Drosophila*[73], Itch in mammals [86]), and on the other hand with eIF3f, a DUB required for Notch activation process (see below [87]). These apparent discrepancies may actually highlight the various roles of Deltex proteins as Notch-fate facilitators. Identifying the targets of Deltex as an E3 ubiquitin ligase would surely help in deciphering these various roles.

Finally, an additional E3 ubiquitin ligase that could have a role in Notch pathway is C-cbl, although it was shown in mammals to promote Notch degradation [88], and in *Drosophila* to control Notch ligand activity [89].

### 4.3. Notch Activation Process

As mentioned before, the importance of Notch internalization and ubiquitination after its ligand activation is still a matter of debate. Nevertheless evidence exists in all systems, that endocytosis of the activated receptor is necessary for efficient signaling [56,90]. In mammals, it has been proposed that activated Notch is monoubiquitinated after its early endocytosis and before its gamma-secretase cleavage [90]. Recently two lines of evidence have strengthened this model: first the identification through an RNAi screen in mammalian cells of a DUB named eIF3f, acting positively on Notch signaling. This DUB targets specifically activated, ADAM-cleaved Notch and allows further Notch processing by the gamma-secretase activity, thus regulating NIC production [87]. Second, another screen, this time performed in *Drosophila*, has also identified several DUBs as regulating the Notch signaling pathway in two classical Notch-dependent differentiation models (differentiation of the sensory organ precursors—the bristles on the dorsal part of the thorax of the flies-, and establishment of the wing dorso-ventral polarity). Interestingly eIF3f came out again in this study [35], reinforcing the hypothesis of a conserved ubiquitination/deubiquitination step targeting activated Notch and necessary for signaling. eIF3f is proposed to be recruited to activated, internalized Notch through Deltex protein [87], reinforcing the model according to which Deltex could have antagonistic functions, nevertheless linked to ubiquitination events, and depending on the localization of the Notch-Deltex pair and on the activation state of the receptor.

Recently, Mdm2 (Murine double minute), another E3 ubiquitin ligase of the RING family known as a main regulator of p53 tumor suppressor protein, has been proposed to play a role in Notch signaling: first by upregulating the ubiquitination of Numb [91], leading to Numb degradation, and thus indirectly to an increase of Notch signaling; second by directly targeting Notch 1, resulting in stabilization and activation of NIC [92]. However another study proposes Notch 4 to be a substrate for Mdm2-mediated ubiquitination and degradation, in a manner inversely proportional to the quantity of p53 protein in the cell [93]. It is thus possible that Mdm2-Numb complexes coordinate the regulation of both the p53 and the Notch pathways.

### 4.4. Ligand Maintenance and Signaling Activity

In addition to controlling the activity of the receptor, ubiquitination processes also affect the ability of the ligands to signal.

As a matter of fact, Delta binding to Notch is not sufficient to induce signal in *Drosophila*, and signaling ensues only if DSL proteins are coexpressed with one of the E3 ubiquitin ligases Neuralized (Neur) or Mindbomb (Mib) [94–98]. These E3 ubiquitin ligases belong to the RING family and are conserved along evolution with gene duplication (see Figure 4 and Table 1). They are able to ubiquitinate Notch ligands *in vitro* and to stimulate their internalization [94,95,99,100]. However the two enzymes use distinct docking sites and catalyze ubiquitination on different lysine residues of the intracellular domain of *Drosophila* Delta. Depending on the context (wing dorso-ventral boundary induction, or sensory organ precursor differentiation), the ubiquitination sites on Delta as well as the required E3 are different [101], in accordance with previous observations that they have distinct developmental functions [97].

Conservation in mammals of the *Drosophila* Delta motifs of interaction with the E3s is good for some (providing a Mib1 interaction platform), but quite bad for Neuralized, suggesting that the molecular roles of these E3s may be different between vertebrates and insects. Contrasting results have been obtained in mammals regarding the function of Neuralized orthologs: on one hand, overexpression experiments have shown that Neur2 is able to stimulate Dll1 internalization, localization into Hrs-positive vesicles or recycling to the apical plasma membrane of polarized cells [102,103], whereas Neur1 would be able to act on Jagged1 [104]. This suggests that Neuralized-like E3 ubiquitin ligases could have a function in the ligand biology, maybe by acting downstream of early endocytosis directed by Mib1. On the other hand, *Neur1*−/−: *Neur2*−/−, as well as *Mib2*−/− mice are normal [105], suggesting that these factors are dispensable or that compensatory mechanisms exist. In contrast, *Mib1* invalidation leads to defects reminiscent of those of *Notch* knockouts: mice die prior to embryonic day 11.5, with pan-Notch defects in somitogenesis, neurogenesis, vasculogenesis and cardiogenesis, Notch target genes expression is reduced, NIC is not produced and Dll1 accumulates at the cell surface [100,105,106], suggesting that Mib1 is the most important E3 in mammals, at least in the embryo. However, invalidation of *Notch* genes or ligand-encoding genes separately also leads to more or less severe phenotypes, because some genes are largely expressed and used (*Notch 1*, *2*, *Dll1*, *4*, *Jag1*), others have more discrete functions (*Notch 3*, *4*, *Dll3*, *Jag2*), and probably because of redundancy [29]. Despite the difficulty in interpreting the phenotypes of the KO mice, all of these factors have physiological and context-dependent roles as Notch receptors and ligands, and the same could be true for the E3 ubiquitin ligases.

Apart from the requirement of E3 ubiquitin ligases activities, what do we know about the relationship between ubiquitination and ligand activity/quantity?

Two phenomena requiring ubiquitination and necessary for the signaling activity of ligands have been described.

#### 4.4.1. Transendocytosis

Ligand endocytosis after contacting the Notch receptor creates a pulling force that promotes receptor proteolysis and directly participates in downstream signaling, a process named transendocytosis (Figure 2) [107–109]. This model has been proposed after the observation of Notch extracellular domain colocalized with Delta in vesicles of wing discs cells of *Drosophila*, in a dynamin-dependent manner [110]. In mammals the same observations were made when coculturing Dll1 (or Jagged1)-expressing cells together with Notch-expressing cells [107,109]. Transendocytosed Notch was visible in Delta-expressing cells next to Notch cells where NIC was detected, showing that indeed this event was necessary to cluster Notch and disrupt the intramolecular interactions that keep it intact and inactive. This physical dissociation of the heterodimer is a prerequisite for proteolysis by ADAM and downstream events of activation. This process was elegantly explored by using optical tweezers that measured the strength of Dll1 interaction with Notch1-Fc beads to about 10pN [108,111].

Upon interaction between a ligand and its receptor, ubiquitination of the ligand is stimulated, allowing the recruitment of the endocytosis machinery. The factor accounting for ubiquitinated ligand recognition is Epsin (Lqf in *Drosophila*), a clathrin-interacting endocytic adaptor that is thought to bind the ligands through its ubiquitin interacting motif (UIM) [108,112–114]. Epsin’s role in ligand-emitting cell is rather complex and requires three epsin functional domains [114]: the ENTH (Epsin-*N*-Terminal Homology) domain binds PIP2 (phosphatidylinositol 4,5-biphosphate), inserts into the plasma membrane and induces membrane curvature. Then the epsin *C*-terminus recruits clathrin directly and also AP-2 and/or Eps15 to eventually promote clathrin-dependent ligand endocytosis. As epsin UIM exhibits a rather weak affinity for ubiquitin or mono-ubiquitinated protein, it is probable that in addition to ubiquitination, other factors or events are necessary to induce ligand internalization.

#### 4.4.2. Recycling of the Ligands

It has been proposed that the ligands should go through a recycling/maturation step before interacting with the receptor (Figure 2) [107,109,110,115]. This recycling is necessary for Dll1 and Dll4 to activate Notch1, and depends on ligand ubiquitination [107,116]. Ubiquitination of Dll1 in this process takes place after endocytosis, and consists in the attachment of a single ubiquitin to one or several positions (mono or multi-ubiquitination) [107]. This is not true for Dll3, which is devoid of any Lysine residues in its intracellular domain, but which is still able to recycle, being however unable to activate Notch 1 [107,117]. Interestingly, a mutant of lysine 613 of murine Dll1, which is located in the putative Mib-interaction motif, is altered in its ability to bind Notch1, but is still able to bind Mib1 and to recycle [118]. The only detected difference with this mutant is the enhancement of the Dll1 fraction associated with lipid rafts. In the assay conditions necessary for using the optical tweezers, *i.e.*, with Notch fused to an Fc fragment and immobilized on beads, neither endocytosis nor recycling strengthen ligand binding to Notch: inhibition of dynamin, Epsin or rab-11-dependent recycling did not significantly affect the value of the rupture force, but instead resulted in increasing Dll1 at the cell surface [111]. According to these data, recycling rather determines ligand level at the cell surface and therefore the signaling intensity.

Collectively these results suggest that inhibiting recycling of the ligands results in lowering Notch-dependent activation. Ligand recycling could affect processing, association with cofactors, appropriate localization, protection from degradation and/or shedding, and/or clustering of the ligands at the cell surface. Ligand localization to specific membrane subdomains is thus an important parameter, maybe directing the recycling pathway, and allowing the ligand to be fully active. In accordance with this hypothesis, Dll1 has been shown to be partly present in rafts [107,118], and *Drosophila* mutants affecting the glycosphingolipids (GSL) biosynthesis present genetic interactions with the Notch signaling pathway by affecting Mib-dependent endocytosis of Delta. In addition, a conserved motif identified in the *N*-terminal extracellular part of Delta, Serrate and their mammalian counterparts, accounts for ligand interaction with GSLs, which are particularly enriched in rafts [119], raising the possibility that direct GSL—protein interactions modulate the endocytosis of Notch ligands. In contrast, Dll4 has been shown to be excluded from raft microdomains in conditions where Dll1 remained partially associated with these fractions [116]. However, recycling, Mib1 association and ubiquitination of Dll4 are still required for Notch 1 uptake and activation by this ligand in T cell differentiation.

A model that conciliates all data would be that Dll1, first addressed to raft subdomains, and Dll4 at the cell surface, require recycling in an ubiquitination-dependent fashion to be eventually clustered or modified at the cell surface. Upon Notch binding, the ligands, bound to the extracellular subunit of Notch, are again ubiquitinated at the cell membrane and then transported by the epsin and clathrin machinery. This internalization event creates the mechanical force breaking the Notch heterodimer, the initiating step of Notch activation.

These results highlight the importance of at least two ubiquitination events undergone by Notch ligands, taking place for the first after early endocytosis, for the second at the cell surface, and possibly catalyzed by the same E3 ubiquitin ligases. Careful studies of the ubiquitination and deubiquitination events undergone by each of the Notch ligands and cofactors will surely give new insights on these intricate mechanisms. Another important phenomenon involving Notch receptors and ligands, called *cis*-inhibition, seems to be independent of ubiquitination of the ligand. This process allows Notch receptor to be inhibited rather than activated by the ligands present within the same cell, helping by this way to restrict receptor activation to specific cells [120].

## 5. Conclusions

A lot remains to be understood linking Notch signaling pathway to ubiquitination and deubiquitination mechanisms. A recent illustration is given by screens performed in the *Drosophila* system and aimed at identifying new regulators of Notch signaling [121,122]. Among modulators affecting all aspects of cell life, ubiquitination and deubiquitination factors were each time identified, and need further studies. Beside its importance as a key signaling pathway, Notch pathway can be seen as a model system where numerous and different types of ubiquitination events (targeting various substrates, with various types of chains, localizations and outcomes) are used.

## Figures and Tables

**Table 1:** Receptors, ligands, E3 ubiquitin ligases and deubiquitinating enzymes (DUBs) of the Notch pathway.

Component function	*Drosophila*	*Caenorhabditis elegans*	Mammals
Receptor	Notch	LIN-12, GLP-1	Notch 1–4

Ligand (DSL)	Delta	APX-1, LAG-2, ARG-2, DSL1-7	Dll 1, 3, 4
	Serrate		Jagged 1, 2

E3 ubiquitin ligase	Mindbomb1–2		Mindbomb 1, 2 (skeletrophin)
	Neuralized		Neuralized 1, 2
	Deltex		Deltex 1–4
	Nedd4, Su(Dx)	WWP-1	Nedd4, Itch/AIP4
	Archipelago	SEL-10	Fbw-7/SEL-10
	d-cbl		Cbl
			Mdm2

DUB	USP12 (CG 7023)		USP12
	eIF3-S5 (CG 9769)		eIF3f
